# Social anxiety in people with facial palsy: The role of fear of negative evaluation and appearance‐fixing behaviour

**DOI:** 10.1111/bjhp.70018

**Published:** 2025-09-02

**Authors:** Jessica A. E. Dring, Emily Reeves, Matthew Hotton

**Affiliations:** ^1^ Oxford Institute for Clinical Psychology Training and Research University of Oxford Oxford UK

**Keywords:** health psychology, social anxiety, social appearance anxiety, visible differencefacial palsy

## Abstract

**Objectives:**

The current study aimed to explore whether people with facial palsy experienced more social appearance anxiety, social anxiety, fear of negative evaluation, and engaged in more appearance‐fixing behaviour than controls without facial palsy. The secondary aim was to investigate whether fear of negative evaluation and appearance‐fixing behaviour were predictive of social appearance anxiety in people with facial palsy.

**Method:**

People with facial palsy (*n* = 78) and people without facial palsy (*n* = 86) completed online questionnaires with measures of social anxiety, social appearance anxiety, fear of negative evaluation, and appearance‐fixing behaviour.

**Results:**

The facial palsy group experienced significantly greater social appearance anxiety, social anxiety, and fear of negative evaluation than the control group; controlling for depression. The facial palsy group also engaged in significantly more appearance‐fixing behaviour than the control group. Further, controlling for depression, fear of negative evaluation from others and appearance‐fixing were both found to be significant positive predictors of social appearance anxiety in the facial palsy group.

**Discussion:**

Findings are consistent with Clark and Well's (1995) cognitive behavioural model of social anxiety. Findings indicate a need for screening and provision for psychological support for social anxiety in people with facial palsy and that cognitions relating to fear of negative evaluation and appearance‐fixing behaviour are potentially useful targets for intervention.

**Statement of contribution**

**What is already known on this subject?**

- Facial palsy can lead to changes in facial appearance and function. This can impact physical and social functioning, as well as psychological well‐being. Qualitative research has highlighted that social appearance anxiety is a key issue for some people with facial palsy. The cognitive behavioural model of social anxiety would predict that fear of negative evaluation and appearance‐fixing safety‐seeking behaviours may play a role in experiences of social appearance anxiety in people with facial palsy. Thus far, there has been limited quantitative research investigating social anxiety and the potential role of cognitive behavioural factors in people with facial palsy.

**What does this study add?**

- This study shows that people with facial palsy experienced greater social anxiety and social appearance anxiety than people without facial palsy.
- People with facial palsy experienced greater fear of negative evaluation and engaged in more appearance‐fixing behaviour than people without facial palsy.
- Fear of negative evaluation and appearance‐fixing behaviour positively predicted social appearance anxiety in people with facial palsy.

## INTRODUCTION

Facial palsy, which occurs following facial nerve damage, can lead to changes in facial appearance and function. An estimated 100,000 individuals in the UK live with facial palsy (Facial Palsy UK, 2012). Facial palsy can significantly impact psychological well‐being, with up to 40% and 31.2% of those with facial palsy living with anxiety and depression respectively (Hotton et al., 2020). In comparison, an estimated 5.9% and 3.3% of the general population have clinical levels of depression and anxiety (McManus et al., 2014). High levels of distress in facial palsy may be in part due to the impact on social and physical functioning (Pattinson et al., 2022). People with facial palsy may have impaired sight and facial function, potentially impacting facial expression, eating, drinking, and speaking (Shindo, 1999). Limited facial animation negatively impacts verbal and non‐verbal communication, which can impact social functioning (Keillor et al., 2002). The impact on social functioning may lead to social isolation and avoidance and consequently a reduction in psychological well‐being (Thompson & Kent, 2001). Social appearance anxiety is a domain of social anxiety related to concern about social situations in which one's appearance may be judged (Hart et al., 2008). Qualitative data positions social appearance anxiety as a key driver of distress in people with facial palsy (Pattinson et al., 2022). However, there is limited quantitative research investigating social anxiety in people with facial palsy.

Research suggests that facial palsy severity does not reflect the level of social functioning and anxiety in people with facial palsy. A systematic review and meta‐analysis found that individuals with the same facial palsy severity may experience social impacts differently and vice versa (Bruins et al., 2021). Further, a systematic review by Hotton et al. (2020) looking at the psychosocial impacts of facial palsy highlighted that social difficulties were a better predictor of anxiety in people with facial palsy than objective facial palsy severity is (Díaz‐Aristizabal et al., 2019; VanSwearingen et al., 1998; VanSwearingen & Brach, 1996). Bogart (2020) also found that people born with facial palsy reported better psychosocial functioning than people with acquired facial palsy, suggesting that the duration of facial palsy may play a role in distress. Collectively, this research suggests that psychological processes, such as cognitive and/or behavioural factors, may play a greater role than aesthetic factors in social functioning and anxiety in facial palsy. Prior research has predominantly measured distress in people with facial palsy rather than investigating the drivers of distress (Pattinson et al., 2022). Therefore, it is important to better understand what factors contribute to social anxiety in people with facial palsy.

Kent and Thompson's (2002) model of distress from disfigurement may help to explain why people with facial palsy may be at higher risk for social anxiety. Kent and Thompson (2002) propose that prior experiences, societal messages and stigma predispose assumptions that one's appearance can single one out as unattractive, defective or at risk of social exclusion or rejection. Individuals with facial difference experience stigma in the form of public stigma (such as experiences of stereotyping, prejudice, and discrimination), structural stigma (societal perpetuation of stigma), stigma by association (stigma towards companions), and internalized self‐stigma (Rasset et al., 2022). Indeed, people with facial palsy report experiencing stigma and judgement towards people with facial palsy have also been demonstrated in research (Bogart et al., 2012; Parsa et al., 2020; Schmidt & Cohn, 2001). Therefore, social situations may trigger assumptions in those with visible difference that others will analyse and judge their appearance, potentially resulting in negative social evaluation (Hamlet et al., 2021). A fear of negative evaluation is defined as a ‘sense of dread associated with being evaluated unfavourably while anticipating or participating in a social situation’ (Weeks et al., 2005, p. 179). Fear of negative evaluation is implicated in social anxiety (Leary, 2015; Voncken et al., 2010). Therefore, Kent and Thompson's (2002) model suggests that those with facial palsy may experience greater fear of negative evaluation, a cognitive factor associated with social anxiety.

Clark and Wells's (1995) model of social anxiety may help us to understand factors that could play a role in social anxiety in people with facial palsy (Clarke et al., 2014). As social situations may trigger assumptions prompting a fear of negative evaluation from others, individuals may perceive social danger, processing themselves as a social object. This leads to focusing attention internally rather than externally on the social event. Consequently, individuals may engage with safety‐seeking behaviours; behaviours enacted to prevent or minimize feared outcomes (Salkovskis, 1988), which affect their social interaction. Those with facial palsy report safety‐seeking behaviours in the form of appearance‐fixing behaviours, such as concealing their face, avoiding smiling to reduce facial asymmetry, and social isolation and avoidance of social situations, such as eating and drinking in public (Norris et al., 2019). The model suggests that safety‐seeking behaviours may then maintain the issue, which could also apply to facial palsy. For example, the interference appearance‐fixing has on one's social interaction may lead to feared responses (e.g., avoidance of smiling being misinterpreted by others) and prevent access to information that challenges negative appraisals about image (Hamlet et al., 2021).

Siemann et al. (2023) found that avoidant coping was associated with social appearance anxiety in people with facial palsy. However, no research has investigated appearance‐fixing behaviour in people with facial palsy. Research has highlighted that appearance‐fixing safety‐seeking behaviours may contribute to the maintenance of social anxiety in people with visible differences, such as the use of wigs for alopecia (Montgomery et al., 2017) and skin camouflage for skin blemishes (Kent, 2002). Therefore, according to Clark and Wells's (1995) model, fear of negative evaluation and safety‐seeking appearance‐fixing behaviours could be key factors in the maintenance of social anxiety in those with facial palsy.

To the best of authors' knowledge, currently only one study has investigated social anxiety quantitatively in people with active facial palsy. Siemann et al. (2023) investigated associations between sociodemographic characteristics, disease‐related factors, broad coping style (problem‐focused, emotion‐focused or avoidant) and social anxiety and social appearance anxiety in 111 people with facial palsy. Avoidant coping (self‐distraction, denial, substance use and behavioural disengagement), female gender, and younger age were predictive of higher social anxiety and social appearance anxiety. The finding that avoidance is predictive of social appearance anxiety is consistent with Clark and Wells's (1995) model. However, Siemann et al. (2023) investigated broad coping styles, rather than specific cognitive and behavioural factors which, according to Clark and Wells's (1995) model, may be relevant to social anxiety in facial palsy, namely, fear of negative evaluation and appearance‐fixing behaviour.

To date, no research has compared levels of social anxiety in people with facial palsy with controls without facial palsy. Comparing levels of distress in people with a visible difference with population reference values is not as valid or reliable as control group comparison (Dalgard et al., 2015). Further, no research has investigated levels of appearance‐fixing behaviour and fear of negative evaluation in people with facial palsy compared with the general population. There is currently limited research testing interventions for psychological distress in people with facial palsy (Hotton et al., 2022). Identifying relevant aspects of the distress people with facial palsy commonly experience using a theoretical underpinning is useful for clinicians' assessment of the most relevant aspects of the problem, to develop a useful formulation and plan an appropriate intervention. Therefore, the current study aims to investigate social appearance anxiety and social anxiety in those with facial palsy and the association between fear of negative evaluation and appearance‐fixing behaviours in social appearance anxiety.

### Aims and hypotheses

The aim of the present study is to test the following hypotheses:

#### Primary hypotheses

1. Individuals with facial palsy will experience greater social appearance anxiety and social anxiety than controls without facial palsy.
2. Individuals with facial palsy will engage in more appearance‐fixing behaviour and experience more fear of negative evaluation than controls without facial palsy.

#### Secondary hypothesis

1. Fear of negative evaluation and appearance‐fixing behaviour will positively predict social appearance anxiety in people with facial palsy.

## METHOD AND MATERIALS

### Design

The current study employed a cross‐sectional, mixed‐model between and within‐group design, using quantitative methodology. Data collection took place between April and May 2023. Ethical approval was obtained from the University of Oxford Central University Research Ethics Committee (CUREC; reference number R85311/RE001). People with facial palsy (facial palsy group) and people without facial palsy (control group) were recruited to compare measures of social anxiety, social appearance anxiety, fear of negative evaluation, and appearance‐fixing behaviour. In addition, a within‐group design was used to identify whether fear of negative evaluation and appearance‐fixing behaviour predicted social appearance anxiety in people with facial palsy.

### Participants

Participants were eligible to take part in the study if they were living in the UK, were over 18 years of age, and either had facial palsy for at least 6 months (facial palsy group) or did not have facial palsy or another condition causing visible difference (comparison group). The participants with facial palsy were recruited from the general population via social media, including the charity Facial Palsy UK's social media (Twitter, Instagram and Facebook). No strategies to target recruitment at specific demographics were used. In addition to recruitment via social media, snowball sampling was used for the control group, whereby participants with facial palsy were encouraged to share the study with others of a similar age and gender. There were no monetary incentives for participating in the study. A priori power analysis conducted using G*Power software concentrated on the primary hypothesis determined that to have a 95% chance (*p* < .05) of detecting a medium effect size (*d* = .5) with power at 80%, the total sample size required would be 128 (*N* = 64 per group; Kang, 2021).

### Patient and public involvement

Prior to recruitment, three women with facial palsy consulted on the measures, information sheet, and debrief sheet. Feedback specified that the materials and survey felt relevant, clear, and accessible. A question around the duration of facial palsy and a progress bar were added following feedback.

### Procedure

Data were collected through an anonymous survey hosted by Qualtrics. Participants accessed the survey via weblinks and QR codes included on study advertisements, which produced the participant information form and consent form before proceeding to the self‐report measures. After submitting their responses, participants were presented with a debrief form, which included signposting information should the questionnaires raise any concerns for participants.

### Measures

A brief questionnaire collected sociodemographic data (age, gender, and ethnicity) and information around the affected side of the face, time since onset, and cause of the facial palsy for the facial palsy group. The standardized measures used, and their detailed specifications and psychometric values, are outlined in Table 1. Depression could potentially explain some variability in social anxiety measures due to social withdrawal associated with depression (Langer & Rodebaugh, 2014) and in fear of negative evaluation due to negative thought processes associated with depression. Research has demonstrated higher rates of fear of negative evaluation in people with depression compared with those without (O'Connor et al., 2002). However, research has found that appearance‐fixing behaviour, as measured by the BISCI‐AF, has not been significantly predictive of depression (Choma et al., 2009). Therefore, a measure of depression was included to control for its potential confounding effects on social anxiety, social appearance, and fear of negative evaluation.

**TABLE 1:** Overview of standardized measures.

Measure	Construct	Administration	Interpretation	Validity evidence
Body Image Coping Strategies Inventory – Appearance‐Fixing Subscale (BICSI‐AF; Cash et al., 2005)	Appearance‐Fixing Behaviour	10‐items rated on a 4‐point Likert scale (0 = definitely not like me to 3 = definitely like me)	Higher total scores indicate more use of appearance‐fixing behaviour	The subscale shows good reliability, validity, and internal consistency in the general population (α = .75–.87; Akram et al., 2021; Cash et al., 2005) and in people with visible difference (α = .86; Egan et al., 2011; Zucchelli et al., 2020, 2022)
The Brief Fear of Negative Evaluation Scale (BFNE; Leary, 1983)	Fear of Negative Evaluation	12‐items rated on a 5‐point Likert scale (1 = not at all characteristic of me to 5 = extremely characteristic of me)	Higher total score indicates greater fear of negative evaluation	The measure shows good internal consistency and reliability in the general population (α = .90; Collins et al., 2005) and in people with visible difference (α = .83–.98; Merz et al., 2018; Mills et al., 2018)
The Social Appearance and Anxiety Scale (SAAS; Hart et al., 2008)	Social Appearance Anxiety	16‐items rated on a 5‐point Likert scale (1 = not at all to 5 = extremely)	Higher total score indicates greater appearance‐related social anxiety	The measure shows good test–retest reliability, internal consistency and divergent factor validity in the general population (Hart et al., 2008; Levinson et al., 2013). It has been used previously in research with facial palsy (Siemann et al., 2023) and has shown good internal consistency in people with visible difference due to burns and systemic scoliosis (α = .96; Ayhan et al., 2021; Mills et al., 2018)
Social Interaction Anxiety Scale and Social Phobia Scale – Short Form (SIAS‐6/SPS‐6; Peters et al., 2012)	Social Anxiety	12‐items rated on a 5‐point Likert Scale (0 = not at all characteristic or true of me to 4 = extremely characteristic or true of me)	Higher total scores indicate greater social anxiety	The measures demonstrate accuracy in measuring social anxiety and good convergent validity (Peters et al., 2012). The SIAS‐6 has been validated in people with visible difference due to scleroderma, showing good convergent validity, internal consistency and reliability (Gholizadeh et al., 2018)
Patient Health Questionnaire (PHQ‐8; Kroenke & Spitzer, 2002)	Depression	8‐items rated on a 4‐point Likert scale ‘over the last 2 weeks’ (0 = not at all to 3 = nearly every day)	Higher total score indicates more severe depressive symptoms	The measure is a validated diagnostic and severity measure for depressive disorders (Kroenke & Spitzer, 2002). The measure shows good internal consistency in people with visible difference due to scleroderma (α = .89; Gholizadeh et al., 2018)
The Facial Disability Inventory – Physical Functioning Subscale (FDI‐PF; VanSwearingen & Brach, 1996)	Facial Function	5‐items rated on a 6‐point Likert scale ‘during the last month’ (0 = usually did with no difficulty to 5 = usually did with no difficulty.)	Higher total scores indicate worse physical functioning and greater facial disability	The measure was developed for people with facial nerve paralysis. It has shown high internal consistency, good construct validity, and responsiveness (VanSwearingen et al., 1998; VanSwearingen & Brach, 1996). The physical function subscale has illustrated good internal consistency (α = .72) and test–retest reliability (intraclass correlation coefficient = .84) in people with facial palsy (Van Veen et al., 2020)

## ANALYSES

Item five from the 16‐item SAAS was missing from all participants' data due to an administrative error prior to data collection. Mean substitution was used because the missing data were unrelated to other study variables and were missing for all participants (Baraldi & Enders, 2010; Eekhout et al., 2012). This was deemed appropriate due to the high level of internal consistency found for this measure within the current study (α = .97 in the facial palsy group and α = .96 in the control group).

### Primary analyses

Depression scores for the facial palsy group (mean rank = 104.73) were statistically higher than for the control group (mean rank = 62.34); *U* = 1620.00, *z* = −5.73, *p* < .001, with a medium effect size of *r* = .45. Therefore, depression was controlled for in analyses regarding the social anxiety measures and fear of negative evaluation. Data were examined to check whether necessary statistical assumptions had been met. Data for fear of negative evaluation and appearance‐fixing behaviour were normally distributed, but data for social anxiety and social appearance anxiety were not. Log transformation was performed on social anxiety scores, resulting in a normal distribution. However, performing appropriate transformations did not improve the distribution for social appearance anxiety. Therefore, a robust ANCOVA was carried out using the WRS2 package in R to determine if there were differences in social appearance anxiety, as this test does not make any parametric assumptions (Mair & Wilcox, 2020; Wilcox, 2022). Two ANCOVAs were employed to determine if there were differences in social anxiety and fear of negative evaluation between groups, controlling for depression. An independent *t*‐test was used to determine if there were differences in appearance‐fixing behaviour rather than an ANCOVA, as there was no theoretical rationale to include depression as a covariate.

### Secondary analyses

This study looked to find associations between fear of negative evaluation and appearance‐fixing behaviour and social appearance anxiety. Therefore, the data for the secondary analysis was also examined to check necessary statistical assumptions for a hierarchical regression, and the assumptions were met. Correlation analysis indicated that age was not correlated with social appearance anxiety, nor was facial functioning and time since onset (see Table S1 for correlation matrix). Therefore, only depression was controlled for in the analysis.

## RESULTS

### Descriptive statistics

A total of 167 participants completed the questionnaires. Three participants from the facial palsy group had acquired facial palsy under 6 months before, so their data had to be removed, leaving a sample of 164 participants (*N* = 78 facial palsy group, *N* = 86 control group). Both samples were majority women (facial palsy group 92% women, control group 93% women). Ages ranged from 19 to 68 years old in the facial palsy group and 23–72 years old in the control group. The average age was 47 years for both groups, and there was no statistically significant difference in age between the groups *t*(162) = −.12, *p* = .45. Regarding ethnicity, 8.1% of the facial palsy group and 17.4% of the control group identified as people of the Global Majority (belonging to Black, Asian, dual‐heritage and/or indigenous to the global south ethnic groups). Regarding the facial palsy group specifically, time lived with facial palsy ranged between 6 months and 61 years, 2 months. There were a range of causes for facial palsy, and the results of the FDI indicated a range of facial disability with scores ranging from 0 to 100. Table 2 provides more detail on sample demographics for descriptive and reliability statistics for the measures of interest.

**TABLE 2:** Sample characteristics.

Demographics	Facial palsy group (*N* = 78)	Control group (*N* = 86)
Gender		
Female	72	80
Male	6	6
Age (mean, *SD*)	47.99 (12.73)	47.76 (11.70)
Ethnicity		
White British, Irish or other White background	71	71
Indian	0	2
Pakistani	3	4
Other Asian background	1	0
Black African	0	2
Black Caribbean	1	1
Other Black background	0	2
Mixed White and Asian	0	1
Mixed White and Black Caribbean	1	1
Other mixed background	0	1
Any other	1	1
Cause of Facial Palsy		
Bell's Palsy	36	
Ramsay Hunt Syndrome	16	
Acoustic Neuroma	8	
Congenital	4	
Tumour	3	
Parotidectomy	3	
Head Injury	1	
Cholesteatoma	1	
Unknown	6	
Affected side of face		
Unilateral left side	40	
Unilateral right side	34	
Bilateral	4	
Facial function (FDI‐physical index; mean, *SD*)	42.24 (28.13)	
Mean years and months since onset (*SD* in months)	13 years 3 months (174.00)	

### Primary hypotheses

A robust ANCOVA was conducted to determine if there were differences in social appearance anxiety between groups, controlling for depression. Four design points (i.e., levels of depression) were generated by R, as design points exceeding 12 did not have ≥12 participants per group (Wilcox, 1997). The robust ANCOVA demonstrated that the facial palsy group consistently had significantly higher levels of social appearance anxiety compared with the control group across each level of depression (see Table 3). This demonstrates that after controlling for depression scores, social appearance anxiety was statistically significantly greater in the facial palsy group versus the control group, *p* < .001, with a large effect size of *δt* = −2.18 (Mair & Wilcox, 2020). An ANCOVA was conducted to determine if there were differences in social anxiety between the groups (see Table 4). After controlling for depression scores, social anxiety was statistically significantly greater in the facial palsy group versus the control group, *F*(1,161) = 29.29, *p* < .001, with a large effect size of partial η^2^ = .15.

**TABLE 3:** Difference in social appearance anxiety between groups at different levels of depression.

PHQ‐8 (depression) level	Facial palsy N (PHQ‐8 score range)	Control N (PHQ‐8 score range)	Mean difference in SAAS	SE	Lower CI	Upper CI	Test statistic	*p*
0	28 (0–6)	51 (0–4)	−27.03	4.16	−38.66	−15.42	6.51	<.001
3	41 (0–9)	67 (0–7)	−26.91	2.96	−34.87	−18.95	9.10	<.001
6	52 (0–12)	47 (3–20)	−24.09	2.80	−31.54	−16.65	8.59	<.001
12	43 (9–22)	14 (8–24)	−25.45	3.44	−35.14	−15.77	7.39	<.001

**TABLE 4:** Adjusted and unadjusted group means and variability for fear of negative evaluation and social anxiety with depression as a covariate and *t*‐test statistics for appearance‐fixing behaviour.

Measure	Group	*N*	Unadjusted		Adjusted	
			*M*	SD	*M*	SE
Fear of negative evaluation	Facial Palsy	78	43.33	8.79	41.39	.92
	Control	86	35.31	8.49	37.08	.87
Social anxiety	Facial Palsy	78	1.15	.44	1.04	.04
	Control	86	.61	.49	.71	.04

		*N*	*M*	SD	*t*	*p*
Appearance‐fixing behaviour	Facial Palsy	78	1.63	.78	4.18	<.001
	Control	86	1.14	.72		

*Note*: Values for Social Anxiety are for the log transformed data.

An ANCOVA was run to determine if there were differences in fear of negative evaluation with depression as a covariate (see Table 5). After controlling for depression scores, fear of negative evaluation was statistically significantly greater in the facial palsy group versus the control group, *F*(1,161) = 10.68, *p* = .001, with a medium effect size of partial η^2^ = .062. An independent *t*‐test was run to determine if there were differences in appearance‐fixing behaviour scores between groups (see Table 4). Appearance‐fixing behaviour scores for the facial palsy group were statistically higher than for the control group, *M* = .49, SE = .12, 95% CI [.26, .72], *t*(162) = 4.18, *p* < .001, with a medium effect size of *r* = .31 (Rosenthal, 1991).

**TABLE 5:** Hierarchical multiple regression analysis predicting social appearance anxiety from fear of negative evaluation and appearance‐fixing behaviour, controlling for depression.

	*b*	*SE b*	△*R* ^2^	*β*	*t*	*p*
Step 1			.253			
Constant	46.93	2.75			17.04	<.001
Depression	1.19	.23		.51	5.20	<.001
Step 2			.645			
Constant	4.36	5.23			.83	.407
Depression	.50	.18		.21	2.81	.006
Fear of negative evaluation	1.00	.14		.58	7.10	<.001
Appearance‐fixing behaviour	3.74	1.57		.19	2.38	.020

All statistical tests for hypotheses 1 and 2 remained statistically significant at Bonferroni corrected confidence level of *p* < .0125 (i.e., correcting for four comparisons).

### Secondary hypothesis

A hierarchical multiple regression was run to determine if fear of negative evaluation and appearance‐fixing behaviour predicted social appearance anxiety in the facial palsy group (see Table 5). Depression was entered in step one as a covariate; then fear of negative evaluation and appearance‐fixing behaviour were added in the final step. Model 1 included depression as the sole predictor variable and was found to explain a significant proportion of variance in social appearance anxiety, *F*(1,76) = 27.06, *p* < .001, adjusted *R* ^2^ = .25. The addition of fear of negative evaluation and appearance‐fixing behaviour to the prediction of social appearance anxiety (Model 2) led to a statistically significant increase in *R* ^2^ of .40, *F*(3,74) = 47.69, *p* < .001, adjusted *R* ^2^ = .65. Together, fear of negative evaluation and appearance‐fixing accounted for 39.7% variance of the model. Examined separately, fear of negative evaluation positively significantly predicted social appearance anxiety (*b* = 1.00, CI 95% [.72–1.29], *p* < .001), as did appearance‐fixing behaviour (*b* = 3.74, CI 95% [.61–6.87], *p* = .02).

## DISCUSSION

The primary aim of this study was to explore whether people with facial palsy experienced more social appearance anxiety, social anxiety, fear of negative evaluation, and engaged in more appearance‐fixing behaviour than controls without facial palsy. The secondary aim of the study was to investigate whether fear of negative evaluation and appearance‐fixing behaviour were predictive of social appearance anxiety in people with facial palsy.

All alternative hypotheses were met. *H*_1_ and *H*_2_: People with facial palsy experienced significantly greater social appearance anxiety, social anxiety, fear of negative evaluation, controlling for depression, and engaged in more appearance‐fixing behaviour than the controls without facial palsy. Medium to large effect sizes were found for these differences. These findings are in line with qualitative research in which people with facial palsy have reported appearance‐related distress, anxiety in social settings, fear of judgement and reactions from others as well as appearance‐checking and fixing actions (Hamlet et al., 2021; Pattinson et al., 2022). It also supports previous research showing high rates of psychological distress and social anxiety in people with facial palsy (Hotton et al., 2020; Krane et al., 2020; Siemann et al., 2023). Finally, *H*_3_: Fear of negative evaluation from others and appearance‐fixing were both found to be significant positive predictors of social appearance anxiety in those with facial palsy. This is in line with research into other visible differences whereby appearance‐fixing behaviour has been associated with social anxiety (Kent, 2002; Montgomery et al., 2017). Findings also align with wider literature around fear of negative evaluation as a risk factor for social anxiety and social appearance anxiety in people without visible difference (Carleton et al., 2011; Clark & Wells, 1995; Levinson et al., 2013). To the best of authors knowledge, no other research has investigated whether appearance‐fixing behaviour and fear of negative evaluation predict social appearance anxiety in people with visible difference.

The study findings are consistent with Clark and Wells's (1995) model of social anxiety, wherein people with facial palsy experience greater fear of negative evaluation from others, which may prompt engagement in safety‐seeking behaviours in the form of appearance‐fixing behaviours. These cognitive and behavioural factors are predictive of social appearance anxiety. Thus, appearance‐fixing behaviours potentially maintain social appearance anxiety by way of preventing the disconfirmation of, and/or contributing to, the fears of negative evaluation. Findings also align with Kent and Thompson's (2002) model of distress in disfigurement and Rasset et al.'s (2022) social stigma model in facial difference, wherein increased fear of negative evaluation in people with facial palsy compared with controls could be indicative of assumptions around negative evaluation due to their visible difference and associated internalized stigma. This was the first study to compare levels of social anxieties in those with facial palsy with controls without facial palsy. A further strength of the study was the use of validated questionnaires which demonstrated excellent internal consistency within this sample.

Interestingly, fear of negative evaluation was a greater positive predictor of social appearance anxiety than appearance‐fixing behaviour. This could suggest that fear of negative evaluation cognitions play a greater role in social appearance anxiety than appearance‐related safety‐seeking behaviour for people with facial palsy. However, there are other potential explanations. First, avoidance has previously been found to predict social appearance anxiety in those with facial palsy (Siemann et al., 2023), which was not measured in this study. Second, questions on the BICSI‐AF, the measure for appearance‐fixing behaviour, predominantly relate to attractiveness. It could be that the fear of negative evaluation was also related to the negative impact of limited facial animation on verbal and non‐verbal communication. Limited ability to smile and effectively express emotion has been identified as a key distressing aspect of facial palsy (Hamlet et al., 2021; VanSwearingen et al., 1999). Research has found that people with severe facial palsy can be perceived as having more negative affect compared with milder facial palsy (Bogart et al., 2014). It could be that the impact of facial palsy on facial expression in social interaction also influences social appearance anxiety and that there are safety‐seeking behaviours related to this that are not captured by the BICSI‐AF. Clark and Wells's (1995) model theorizes that people with social anxiety may hold excessively high standards for social performance, which could be exacerbated in people with facial palsy considering the impact of limited facial animation. Future research could investigate the relative importance of avoidance and appearance‐fixing behaviour, both relating to attractiveness and to facial animation, in people with facial palsy. To the best of authors knowledge, there is currently not a validated measure that captures appearance‐fixing behaviour related to concerns about limited facial animation.

### Clinical implications

These findings highlight the importance of screening and provision of support for the psychological impact of living with facial palsy. Further, this study found no association between self‐reported facial function and social appearance anxiety, social anxiety, fear of negative evaluation, or appearance‐fixing behaviour. This adds to other research which has found a limited association between the severity of facial palsy and distress (Bruins et al., 2021; Hotton et al., 2020). Instead, findings emphasize the importance of cognitive behavioural factors in the development and maintenance of social appearance anxiety in facial palsy and thus the opportunity for psychological intervention.

Cognitions relating to fear of negative evaluation from others and appearance‐fixing behaviour may be helpful targets for interventions, such as cognitive behavioural therapy (CBT; Clarke et al., 2014). Some cognitions may hold some degree of objective reality for people with facial palsy (e.g., ‘people will stare’). Therefore, cognitive restructuring techniques should not challenge the reality of such thoughts, rather help individuals hold more balanced and compassionate beliefs (e.g., ‘people staring does not mean they are judging me negatively’). Behavioural experiments can also be tailored to test the usefulness of alternative non‐verbal communication strategies to facial animation. For example, tone of voice, verbal expression, or eye contact. It is also important to consider that appearance‐fixing behaviours can be adaptive for some people with visible difference. For example, choosing to apply makeup in ways that enhance symmetry, not to conceal out of fear of judgement, but to simply feel more confident in oneself. Therefore, it is crucial to distinguish between behaviour that serves to maintain unhelpful cognitions and that which helps to improve confidence to engage in social activity (Clarke et al., 2014). Acceptance and commitment therapy (ACT) may also be an effective alternative approach to CBT, due to its focus on detaching from thoughts rather than challenging their reality and on engagement with valued activity regardless of the presence of difficult thoughts and emotions.

There is currently inequity of access to specialist facial palsy services across the UK, particularly regarding psychological support (Facial Palsy UK, 2021; Kilshaw et al., 2016). There is some initial research illustrating that CBT‐based self‐help psychology guides can help with appearance‐related distress in people with facial palsy, which have been made freely available (Hotton et al., 2022). However, it is important to consider the role of societal attitudes in the increased fear of negative evaluation that people with facial palsy experience. In addition to improved provision of psychological interventions for people with facial palsy, it is crucial that there are societal shifts regarding attitudes and stigma around conditions that cause visible difference, such as facial palsy. This highlights the need for awareness campaigns to reduce public stigma around facial palsy.

### Limitations

A limitation of the study was missing data from one item of the 16‐item Social Appearance Anxiety Scale. Cronbach's alpha calculations revealed excellent internal consistency for the SAAS for both groups, which supports the acceptability of mean substitution for the missing data, including with the exclusion of the missing item (α = .965 in the facial palsy group and α = .956 in the control group). A shorter domain general scale for fear of negative evaluation was used for this study to measure generalized fear of negative evaluation in keeping with the general model of social anxiety and to balance participant demand. However, future research may benefit from also using the fear of negative appearance evaluation scale (Lundgren et al., 2004). Further, the results of this study were cross‐sectional; thus, causal inferences cannot be made. However, this study represents a starting point, indicating that investigating the role of fear of negative evaluation and appearance‐fixing behaviour in the development of social appearance anxiety using experimental and longitudinal approaches to examine causal relationships as well as assessing intervention effectiveness would be helpful future research directions.

Another limitation was the large gender bias in the sample. Both groups were composed of 92%–93% women. It is possible that there would be gender differences if there were a greater representation of men. Siemann et al. (2023) found that female gender was associated with higher levels of social appearance anxiety in people with facial palsy. This could potentially be due to gender differences in cultural norms around appearance ideals (Grogan, 2010). The facial palsy literature has mixed findings regarding gender and social functioning and distress (Fu et al., 2011; Hotton et al., 2020; Lee et al., 2007; Leong & Lesser, 2015; Volk et al., 2016). Unfortunately, this study cannot add to this literature and the results of this study cannot be generalized to men. Future research should investigate whether there are gender differences in fear of negative evaluation and appearance‐fixing behaviour in people with facial palsy. Research suggests that adverts tailored to men with male images and concise text and encouraging women to invite male friends are strategies that may increase male recruitment, which future research could consider implementing (Ryan et al., 2019).

Further, 18% of the UK population identify as people of the Global Majority (Office for National Statistics, 2021). In the current study, 17.4% of the control group identified as people of the Global Majority, compared with only 8.6% of the facial palsy group, which is not representative of the UK general population. Considering the differences across cultures regarding the importance placed on appearance and attractiveness (Anderson, 2019), future research could investigate cultural influence on social appearance anxiety in facial palsy. Finally, snowball sampling was an element of recruitment, wherein participants with facial palsy were asked to share the study with someone of a similar age and gender. It is not possible to determine how many participants were recruited this way, but this may have introduced bias.

## CONCLUSION

This study suggests that people with facial palsy experience greater social appearance anxiety and social anxiety than the general population and is the first study to demonstrate this with a comparison to controls without facial palsy. Findings suggest that people with facial palsy also experience more fear of negative evaluation and engage in more appearance‐fixing behaviour than the general population, both of which were found to be positive predictors of social appearance anxiety in people with facial palsy. Unfortunately, there were not enough men in the present study for findings to be generalizable to men with facial palsy. Nonetheless, the findings indicate the importance of screening and provision of psychological support to people with facial palsy and fear of negative evaluation and appearance‐fixing behaviour as potential targets for intervention.

## AUTHOR CONTRIBUTIONS

**Jessica A. E. Dring:** Conceptualization; investigation; writing – original draft; methodology; data curation; formal analysis; validation; visualization; project administration; writing – review and editing. **Emily Reeves:** Conceptualization; methodology; supervision; validation; writing – review and editing. **Matthew Hotton:** Conceptualization; methodology; supervision; validation; writing – review and editing.

## Supporting information


Table S1:

## Data Availability

The data that support the findings of this study are available on request from the corresponding author. The data are not publicly available due to privacy/ethical restrictions.
