# Supplementary material for: Social anxiety in people with facial palsy: The role of fear of negative evaluation and appearance‐fixing behaviour
Source: Br J Health Psychol. 2025 Sep 2;30(3):e70018. doi: 10.1111/bjhp.70018 (PMC12402973; doi:10.1111/bjhp.70018)
Supplement: Supplementary file 1 — Table S1: [file BJHP-30-0-s001.docx]

**Supplementary Material: Correlation Matrix**

Table 1

*Spearman’s rho Correlation Matrix of Variables of Interest for Facial Palsy Group*

|  | 1 | 2 | 3 | 4 | 5 | 6 | 7 | 8 |
| --- | --- | --- | --- | --- | --- | --- | --- | --- |
| 1 Age | - |  |  |  |  |  |  |  |
| 2 Facial-function | -.014 | - |  |  |  |  |  |  |
| 3 Time since onset (months) | .107 | .043 | - |  |  |  |  |  |
| 4 Depression | -.157 | -.226* | -.026 | - |  |  |  |  |
| 5 Fear of Negative evaluation | -.147 | -.014 | -.066 | .414** | - |  |  |  |
| 6 Appearance-fixing behaviour | -.053 | -.025 | .165 | .374** | .485** | - |  |  |
| 7 Social appearance anxiety | -.144 | -.134 | .080 | .547** | .763** | .539** | - |  |
| 8 Social anxiety | -.196 | -.164 | .023 | .573** | .627** | .398** | .813** | - |

*Note. *p<0.05, **p<.001 (two tailed). The results marked ** remain significant at Bonferroni corrected confidence level of p<0.002 (i.e. correcting for 28 comparisons).*
